# Urofaecal glucocorticoid metabolite concentrations in African penguin (*Spheniscus demersus*) chick populations experiencing different levels of human disturbance

**DOI:** 10.1093/conphys/coab078

**Published:** 2021-09-14

**Authors:** Juan Scheun, Rebecca J Miller, Andre Ganswindt, Lauren J Waller, Lorien Pichegru, Richard B Sherley, Gavin W Maneveldt

**Affiliations:** 1 Department of Life and Consumer Sciences, University of South Africa, Roodepoort, 1724, South Africa; 2 Mammal Research Institute, Department of Zoology and Entomology, University of Pretoria, Pretoria, 0081, South Africa; 3 National Zoological Garden, South African National Biodiversity Institute, Pretoria, 0002, South Africa; 4 Department of Biodiversity and Conservation Biology, University of the Western Cape, Bellville, 7535, South Africa; 5 Southern African Foundation for the Conservation of Coastal Birds (SANCCOB), Table View, 7441, South Africa; 6 DST-NRF Centre of Excellence at the Percy FitzPatrick Institute of African Ornithology and Institute for Coastal and Marine Research at the Nelson Mandela University, Port Elizabeth, 6031, South Africa; 7 Centre for Ecology and Conservation, University of Exeter, Penry Campus, Penryn, Cornwall, TR10 9FE, UK

**Keywords:** stress physiology, glucocorticoids, ecotourism, chicks, body condition, African penguins

## Abstract

Despite the importance of ecotourism in species conservation, little is known about the industry’s effects on wildlife. In South Africa, some African penguin (*Spheniscus demersus*) colonies have become tourist attractions. The species is globally endangered, with population sizes decreasing over the past 40 years. As African penguin chicks are altricial and unable to move away from anthropogenic stressors, it is important to evaluate the effect of tourist activities on baseline glucocorticoid levels as a measure of potential disturbance. Chicks at three study sites within two breeding colonies (Robben Island, Stony Point), with varying levels of exposure to tourism (low/moderate/high) were monitored. Urofaecal samples were collected to determine urofaecal glucocorticoid metabolite (ufGCM) concentrations as an indication of baseline stress physiology. Morphometric measurements were taken to compare body condition between sites. Penguin chicks experiencing low, infrequent human presence had significantly higher mean (± standard deviation) ufGCM levels [1.34 ± 1.70 μg/g dry weight (DW)] compared to chicks experiencing both medium (0.50 ± 0.40 μg/g DW, *P* = 0.001) and high levels of human presence (0.57 ± 0.47 μg/g DW, *P* = 0.003). There was no difference in chick body condition across sites. These results suggest that exposure to frequent human activity may induce habituation/desensitization in African penguin chicks. Acute, infrequent human presence was likely an important driver for comparatively higher ufGCM levels in chicks, though several other environmental stressors may also play an important role in driving adrenocortical activity. Nevertheless, as unhabituated chicks experiencing infrequent anthropogenic presence showed significantly higher ufGCM levels, managers and legislation should attempt to minimize all forms of activity around important breeding colonies that are not already exposed to regular tourism. Although the results of this study are crucial for developing enhanced conservation and management protocols, additional research on the long-term effect of anthropogenic activities on African penguin physiology is required.

## Introduction

Globally, ecotourism is often hailed as a crucial tool for species conservation. It raises revenue through the non-consumptive use of wildlife (Pegas and Castley, 2014; Shannon *et al.,* 2017; Steven *et al.,* 2013) and awareness of environmental issues (Emerton *et al.,* 2006). However, as the industry continues to grow, the expansion of tourist activities into natural, unaltered areas often results in largescale habitat degradation and loss, placing additional pressure on local biodiversity (Rehnus *et al.* 2014). As a result, wildlife species have to respond (behaviourally/physiologically) not only to predictable environmental change, such as seasonal climate change and resource availability, but also to unpredictable human activities within their immediate surroundings (Gaynor *et al.,* 2018). The activation of anti-predator behaviours in response to human activities and natural predators can have direct fitness costs to a population (Amo *et al.,* 2006), so behavioural observations are often collected to determine the cost of human activities to wildlife populations (Bateman and Fleming, 2017; Lowry *et al.,* 2013; Thiel *et al.,* 2008). However, wildlife populations may have little to no observable behavioural reaction in response to human activities, but still produce a physiological response if those activities are perceived as stressors (Ellenberg *et al.,* 2006). Therefore, monitoring the stress physiology of a species directly is a more appropriate way to assess the impact of human activities on wildlife.

Glucocorticoid (GC) levels are generally accepted as a robust proxy of the physiological stress experienced by wildlife populations (Wikelski and Cooke, 2006). Individuals encountering environmental challenge will increase their GC secretion as an adaptive response in order to restore homeostasis (Busch and Hayward, 2009; Romero, 2002). However, the chronic increase of GC concentrations can result in (i) elevated baseline GC levels and (ii) several deleterious effects including immune and reproductive suppression, as well as metabolic alteration (Busch and Hayward, 2009; Webster Marketon and Glaser, 2008; Whirledge and Cidlowski, 2010). Where an animal is unable to cope with environmental challenges, an increase in baseline GC levels will reallocate resources from other activities, such as reproduction and body maintenance. This process, known as the ‘Cort-Fitness Hypothesis’, may result in reduced fitness (Bonier *et al.,* 2009a; Bonier *et al.,* 2009b). However, the support for the Cort-Fitness Hypothesis is not universal and highly context specific, with life history stage being an important determinant (Bonier *et al.,* 2009b; Vitousek *et al.,* 2019). As a result of the important role of sustaining baseline GC levels in maintaining individual and population health and fitness, it is imperative that research be conducted to assess baseline GC levels in wildlife populations facing anthropogenic stressors and how these baseline GC levels might change in response to these stressors.

Non-invasive endocrine monitoring, via the collection and analyses of faeces and urine, provides an ideal method for GC quantification in wild animals above the traditionally used blood collection and analysis (Behringer and Deschner, 2017; Keay *et al.,* 2006). First, non-invasive monitoring techniques minimize the need for prolonged human–animal interaction, thus minimizing any additional potential stress-related feedback (Romero and Reed, 2005), while providing a method that ensures easy, long-term sample collection and analysis (Kersey and Dehnhard, 2014). Secondly, faecal and urine hormone metabolite values are less affected by episodic fluctuations of hormone secretions, as circulating hormone levels within the bloodstream accumulate within the faeces and urine over an extended period of time (McEwen and Wingfield, 2003). Several studies have implemented this method to monitor the stress physiology of penguin species in response to tourist activities in particular, including little penguin (*Eudyptula minor*; Chiew *et al.,* 2019), gentoo penguin (*Pygoscelis papua*; Lynch *et al.,* 2019) and African penguin (*Spheniscus demersus*; Scheun *et al.,* 2020). However, such research has focused largely on the stress physiology of adults within the captive environment, with little to no consideration for penguin chicks, or for the determination of baseline GC levels. As penguin chicks are altricial and thus unable to alleviate any stress experienced by escaping the immediate area, reoccurring stressors may result in chronically elevated baseline GC concentrations (Walker *et al.,* 2005). Such exposure to elevated GC levels has been shown to result in physiological and behavioural change, as well as increased susceptibility to future stressors (Auperin *et al.*, 2008; Kaufman *et al.*, 2007; Teicher *et al.*, 2003). Monitoring adrenocortical activity in penguin chicks in response to tourism activity has only been done in Magellanic penguins (*Spheniscus magellanicus*), albeit through the collection and analysis of blood samples (Walker *et al.,* 2005); newly hatched Magellanic chicks had a significantly higher adrenocortical response to visitation than their control counterparts. However, such a response was not fixed and the physiological response changed as chicks matured (Walker *et al.,* 2005). As a result of the limited research, as well as a possible change in response to tourism as chicks mature, additional research on the topic is needed to determine the physiological effect that ecotourism can have on penguin chicks.

The African penguin is a species endemic to the coastline and islands of southern Africa (Shelton *et al.,* 1984). Marine predators, like the African penguin, are important drivers of trophic stability (Heithaus *et al.,* 2008), while being robust sentinels of habitat quality (Boersma, 2008). As such, conservation efforts of all penguin species are of utmost importance (Ropert-Coudert *et al.,* 2019). Although once abundant throughout its natural range, population sizes have decreased by 72% from 63 000 to ~ 17 000 breeding pairs in the 40 years between 1979 and 2019 (Sherley *et al.,* 2020). As a result, the species is classified as Endangered by the International Union for Conservation of Nature (Birdlife International, 2020). Factors underlying the decline are largely human-driven and include pollution, habitat degradation, overfishing of preferred prey types, disease and climate change (Crawford *et al.,* 2000; Crawford *et al.,* 2011; Frost *et al.,* 1976; Sherley *et al.,* 2017). In addition to this, African penguins face direct contact with humans, especially through tourism activities. There is currently limited information available on the possible effect of such interactions. The only other study to look at the effect of anthropogenic activities on African penguins at different breeding localities (Pichegru *et al.,* 2016) focused on the behavioural response of adults, which could move away from possible stressors. Additionally, that study lacked the inclusion of any physiological markers of stress. Thus, the aim of this study was to examine the relationship of human presence and urofaecal glucocorticoid metabolite (ufGCM) concentrations, as a measure of baseline GC levels, in African penguin chicks. We implemented non-invasive endocrine monitoring at three field sites with varying tourist pressure: two sites at a colony frequently visited by tourists (Stony Point), but contrasting in pressure levels (i.e. medium and high), and one site on an island where human pressure was low (Robben Island), both in the Western Cape Province, South Africa.

## Material and methods

### Study site and animals

The study was conducted between July and October 2017 with the approval of the University of the Western Cape (AR17/5/1) and the University of Exeter (2017/1594) ethics committees.

#### Stony Point

The Stony Point African penguin colony (34.3741° S, 18.8917° E) is located next to a residential area in Betty’s Bay, South Africa. The site was first colonized by the African penguin in 1982, becoming one of the largest breeding colonies in South Africa (Sherley *et al.,* 2020; Underhill *et al.,* 2006). The colony is a popular ecotourist attraction, with an average of 75 000 visitors annually (CapeNature, unpubl. data). Although the colony is not directly accessible to humans, visitors can come into close proximity (1–2 m) to the African penguins, including their nest sites, via a fenced boardwalk (defined as the disturbed study site and termed ‘*SPH*’). As a result, the level of anthropogenic presence at *SPH* is defined as frequent and high. A small section of the same colony is shielded from tourists and is only accessible to reserve staff and researchers (termed *‘SPM’*). The presence of noise pollution from adjacent human activities (boat traffic, residential properties, tourist activities, etc.) does, however, result in the population experiencing frequent human disturbance, though less than at *SPH*. As such, *SPM* is defined as experiencing a medium level of anthropogenic presence. CapeNature (Conservation Authority for the province) conservation and research staff enter both the *SPH* and *SPM* breeding sites every 2–7 days for management and data collection purposes. For this study, chicks were monitored at both sites.

#### Robben Island

Similar to the Stony Point colony, the African penguin colony at Robben Island (33.8076° S, 18.3712° E) in Table Bay, South Africa, was recolonized in 1983 after an ~180-year absence of the species at the study site (Crawford *et al.,* 2006). In total, 320 000 tourists travel to the island annually to visit several historical landmarks (Cape Town Tourism, 2019). However, the main penguin breeding areas are off-limits to visitors, with 1 nautical mile management zone around the island, which limits boat traffic in the vicinity. Similar to Stony Point, conservation and research staff enter some parts of the penguin colony every 2–7 days to collect data on breeding success and for other management purposes (e.g. Sherley *et al.,* 2014a). As such, the penguin populations here experience a lower level of anthropogenic presence than those at Stony Point. For this study, to limit additional disturbance, data were collected from chicks in the areas regularly visited by conservation and research staff (termed ‘*RIL*’).

### Urofaecal collection

In birds, both urine and faeces are excreted in unison through the cloaca and termed as urofaeces (Klasing, 2005; Scheun *et al.,* 2021). Since it appears quite difficult to reliably separate the two matrices without cross-contamination (Scheun *et al.* 2020), the entire sample was collected for analysis. During the collection period, researchers collected a single sample from each chick at the respective study sites. Samples were collected from 14 July to 10 October 2017 (Table 1) during routine chick body condition assessments, thus minimizing the need for additional human–chick interaction. During the assessment, chicks were held above a clean plastic container and all excreted urofaeces was collected, before being transferred into pre-labelled collection tubes. All samples were frozen at −4°C shortly after collection and subsequently transported on dry ice to the Endocrine Research Laboratory, University of Pretoria, South Africa, for hormone analysis.

**Table 1:** The sample collection dates for each study site

Study site	Sample collection dates (2017)
*RIL*	9 August, 17 August, 18 September, 10 October
*SPH*	17 July, 3 August, 21 August
*SPM*	14 July, 3 August, 21 August

### Chick condition index and chick grouping

During handling, researchers used a spring scale to determine individual chick mass (±10 g) and a vernier calliper to measure total head length (±1.0 mm) from the tip of the bill to the back of the skull (Lubbe *et al.,* 2014). For each individual the observed mass $({M}_O)$ and total head length (${H}_O$) measurements were used to calculate the chick body condition index (CCI) using the BCI4 modified Veen index method following Lubbe *et al.* (2014):

$$\begin{align*} CCI&=\left({M}_O-{M}_{max}\right)/\left({M}_{max}-{M}_{min}\right), \\{M}_{min}&=-2472.1629+42.4157\times{H}_O,\\{M}_{max}&=-3499.0741+60.1852\times{H}_O.\end{align*}$$

Here the constants for ${M}_{max}$ and ${M}_{min}$ were derived from the intercept and slope coefficients of 0.95 (${M}_{max}$) and 0.05 (${M}_{min}$) quantile regression fits between total head length and mass from 125 chicks that all fledged on Robben Island in 2004 (Lubbe *et al.,* 2014). The condition of an individual chick is described as the proportion of the distance between the upper and lower quantiles for a chick of a given total head length. The result is a relative index appropriate for calculating the average condition of a group of chicks at a given time, or for comparisons between colonies. Chicks with $0< CCI<1$ have a body condition between the 0.05 and 0.95 quantiles at Robben Island in 2004, $CCI=0.5$ describes the median body condition in the 2004 cohort, and chicks with $CCI<0$ have a reduced probability of surviving to fledging (Lubbe *et al.,* 2014; Morten *et al.,* 2017).

### Urofaecal steroid extraction

The collected urofaecal samples were lyophilized, pulverized and sieved through a thin mesh to remove undigested materials (Fieß *et al.,* 1999). Subsequently, 0.050–0.055 g of urofaecal powder was extracted with 1.5-ml of 80% ethanol by vortexing for 15 minutes at room temperature. Following centrifugation for 10 minutes at 1500 *g*, the supernatant was decanted into microcentrifuge tubes and stored at −20°C until further analysis (Ganswindt *et al.,* 2002).

### Steroid analysis

Urofaecal extracts were analysed with an 11-hydroxyaetiocholanolone assay previously established for the African penguin (Anfossi *et al.,* 2014; Ozella *et al.,* 2015). Here, gut passage time and delay to peak ufGCM levels following a stressor was determined to be 7–10 hours (Anfossi *et al.,* 2014; Ozella *et al.,* 2015); thus, the handling of African penguin chicks in order to collect samples would have no influence on the observed ufGCM levels. Details of the utilized assay, including antibody cross-reactivities, are given by Quillfeldt and Möstl (2003). The sensitivity of the enzyme immunoassay (EIA) was 9 ng/g dry weight (DW). The intra- and inter-assay coefficient of variation, determined by repeated measurements of high- and low-value quality controls, were 6.33% and 6.64%, as well as 5.10% and 7.71%, respectively. Serial dilutions of extracted samples gave a displacement curve that was parallel to the respective standard curve (relative variation in the slope of the trend lines, < 5%).

### Data analysis

In total, 105 urofaecal samples were collected during the study period *(SPH* = 37*, SPM* = 43; *RIL* = 25)*.* Data exploration was conducted following the protocols described by Zuur *et al.* (2010). The assumption of normally distributed data were checked by examining probability plots and conducting a Shapiro–Wilk test for the ufGCM (W = 0.48, *P* < 0.001) and CCI data (W = 0.995, *P* = 0.957). As the ufGCM data were not normally distributed, data were log_10_ transformed to meet the normality assumption. In order to determine whether CCI and varying levels of anthropogenic pressure (low/medium/high) were significantly related to ufGCM levels in African penguin chicks, a general linear model (GLM) was performed with both CCI and anthropogenic pressure as explanatory variables. Only anthropogenic pressure was significantly related to ufGCM levels in this model (see results below). To support the findings of the GLM, a one-way analysis of variance (ANOVA) was conducted to determine if a difference in ufGCM concentration existed between sites (according to the Marginality rule; Crawley, 2014). Subsequently, a *post hoc* Tukey Honest Significant Difference (HSD) test was performed. Finally, we conducted an ANOVA to determine whether CCI differed between the different study sites. All data and graphical analyses were conducted using R (R Core Team, 2019). Data are presented as means ± standard deviation and were considered statistically significant at *P* < 0.05.

## Results

**Figure 1:**
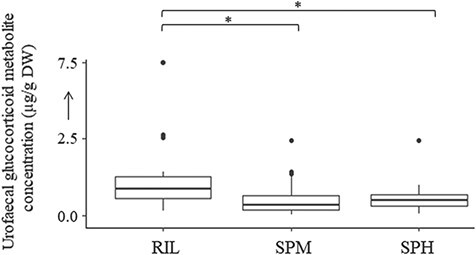
Boxplot (median, 25% percentile, 75% percentile) of ufGCM concentrations (μg/g DW) at each of the three study sections: Robben Island low disturbance (*RIL*), Stoney Point medium disturbance (*SPM*) and Stoney Point high disturbance (*SPH*). Significance between groups is indicated by ^*^.

The GLM analysis indicated that anthropogenic pressure, and not CCI, was significantly related to ufGCM concentrations in African penguin chicks (R^2^ = 0.166, F_(3,101)_ = 7.919, *P* < 0.001; Table 2). The ANOVA analysis conducted in line with the marginality rule supported the GLM results, indicating that differences in anthropogenic pressure was a significant driver of ufGCM levels (F_(2,102)_ = 11.4, *P* < 0.001). The resulting Tukey HSD *post hoc* test showed that chicks from the *RIL* site (1.34 ± 1.70 μg/g DW) had significantly higher ufGCM levels compared to chicks from both the *SPM* (0.50 ± 0.40 μg/g DW, *P* = 0.001) and *SPH* (0.57 ± 0.47 μg/g DW; *P* = 0.003, Fig. 1) sites; there was no significant difference in ufGCM levels between *SPM* and *SPH* sites (*P* > 0.05). There was no statistical difference in CCI across the three sites (F_(2,102)_ = 0.947, *P* = 0.391).

**Table 2:** The coefficient estimates, standard errors (SE), *t*-values, *P*-values and 95% confidence intervals of all predictor variables in the generalized linear model for log_10_ ufGCM concentrations in African penguins at the three study sites

	Coefficient estimate	SE	*t*-value	*P*-value	Confidence intervals	
					2.5%	97.5%
Intercept	−0.298	0.071	−4.212	< 0.001	−0.438	−0.158
CCI	−0.094	0.096	−0.983	0.327	−0.286	0.097
Pressure: *RIL*	0.303	0.091	3.308	**0.001**	0.121	0.484
Pressure: *SPM*	−0.130	0.080	−1.627	0.107	−0.288	0.028

## Discussion

This is the first study quantifying ufGCM as a proxy of the baseline stress physiology of African penguin chicks experiencing different levels of anthropogenic pressure. Our results showed that chicks at Robben Island, experiencing low levels of human pressure, had significantly higher baseline ufGCM levels than their counterparts at Stony Point experiencing medium or high (but regular) levels of human pressure. This is in contrast to several studies on avian species that have shown low GC concentrations in areas with limited to no human presence, including in the capercaillie (*Tetrao urogallus*, Thiel *et al.,* 2005), the American kestrel (*Falco sparverius*, Strasser and Heath, 2013) and the yellow-eyed penguin (*Megadyptes antipodes*, Ellenberg *et al.,* 2007).

Frequent exposure to human activities can lead to a decrease in hypothalamic-pituitary-adrenal (HPA) responsiveness and thus low baseline GC levels (Kleist *et al.,* 2018; Strasser and Heath, 2013), which could account for the findings in this study. A decrease in adrenocortical activity may result from habituation, desensitization, exhaustion and/or life history stage (Cyr and Romero, 2009). Chiew *et al.,* (2019) showed that a change in visitor distance had no effect on the stress physiology of little penguins in captivity, likely due to habituation. Although only assessed behaviourally, African penguins have been shown to be more tolerant of human approach in colonies experiencing high levels of visitor exposure (Pichegru *et al.,* 2016). Such tolerance and habituation could potentially lead to lower baseline ufGCM levels; however, it is still not known which behavioural indices can be linked to stress physiology in this species. Additionally, although an organism may perceive the presence of humans as a threat, chronic exposure can result in the desensitization of adrenocorticotropic hormone responsiveness, and thus the reduction of the resultant stress response mechanism and baseline GC levels (Aguilera, 1994). The chronic hyperactivation of the HPA axis in response to a repeated threat can also lead to individual exhaustion and a lower baseline ufGCM level (Selye, 1946). Finally, season or life history stage can be an important parameter in defining the baseline stress physiology and response of an organism to stressors (Bonier *et al.,* 2009b; Vitousek *et al.,* 2018).

Any one or all of the factors discussed above may have contributed to the lower baseline ufGCM levels observed in the African penguin chicks at Stony Point experiencing medium to high levels of anthropogenic activity. It must be noted that although low GC levels are often perceived as ideal in wildlife populations, suppression (desensitization/exhaustion/habituation) of the HPA axis can lead to various deleterious effects on individual survivability. Firstly, as GCs are important drivers of energy mobility, HPA suppression may lead to the inability of an individual to meet energy requirements, which directly influences growth and development (MacDougall-Shackleton *et al.,* 2019; Schreck, 1993). Additionally, a change in HPA function can lead to alterations in animal ecology, including feeding and mating behaviour, as well as increased predation risk (Busch and Hayward, 2009; Goutte *et al.,* 2011). Further long-term research is required, monitoring several parameters in birds from newly hatched chicks until adulthood, to determine which of the four parameters discussed above could be responsible for the difference in baseline ufGCM levels observed between study populations. Although human presence is a factor that likely drives adrenocortical activity in African penguin chicks, several additional factors might play an important role in this regard and should be studied in future research. Collecting such information will assist in determining the possible long-term effects this may have on chick survival and population demography.

In contrast, chicks from Robben Island that experienced little to no human presence had significantly higher ufGCM levels than those from Stony Point that experienced frequent medium to high levels of human presence. Although close tourist proximity to penguins at the *RIL* site is not possible due to the colony location, anthropogenic factors may still be responsible for elevated baseline ufGCM concentrations in the unhabituated African penguin chicks at the site. Similarly, Berger *et al.* (2007) showed that younger, unhabituated animals have significantly higher baseline GC levels in areas with novel or occasional stressors. Infrequent, distant boat traffic, visitor presence on the island or activities by researchers may be sufficient enough to lead to higher adrenocortical activity and an elevated ufGCM baseline. For example, a human moving to within ~150 m of a nesting site was enough to activate a physiological stress response in unhabituated Humboldt penguins (Ellenberg *et al.,* 2006). Noise has also been shown to activate a behavioural and physiological stress response in several marine and terrestrial birds, including greater sage-grouse (*Centrocerus urophasianus*, Blickley *et al.,* 2012) and crested tern (*Sterna bergii*, Brown, 1990). It should be noted that although elevated baseline ufGCM levels can lead to a decrease in fitness according to the Cort-Fitness Hypothesis, several studies have shown that elevated GC baseline levels can be advantageous. Specifically, such elevation can lead to enhanced post-fledgling survival, in contrast to the Cort-Fitness Hypothesis, through increased locomotor activity and foraging (Rivers *et al.,* 2012). The results of this study found no difference in CCI levels between populations, suggesting that baseline GC levels were not responsible for a decrease in body condition. Furthermore, the relationship between baseline GC levels and fitness depends on the life history stage (breeding/migration, etc.) of a species and should be taken into consideration when monitoring baseline GC levels (Bonier *et al.,* 2009b; Vitousek *et al.,* 2018; Vitousek *et al.,* 2019). Additional research on the *RIL* population is required to establish (i) potential drivers of adrenocortical activity, as well as (ii) the effect of elevated baseline GC concentrations on African penguin chick fitness and long-term survival. Furthermore, although human activity at the area of medium disturbance (*SPM*) does not involve direct visitor pressure (e.g. tourists), frequent, indirect pressure such as boat traffic and chronic urban noise could similarly have led to chick habituation/desensitization and to a decrease in the hyperactivation of the HPA axis. This suggestion, however, needs additional research to confirm.

Aside from direct human activity, several other factors might be responsible for the difference in baseline ufGCM levels of chicks from the two islands. The Robben Island penguin colony has been shown to be impacted by food availability (Campbell *et al.,* 2019), with adult and juvenile survival declining along with prey availability (Sherley *et al.,* 2014b, 2017). A decrease in resource availability and intake can lead to altered behaviour, as well as elevated energy expenditure and physiological stress (Bauer *et al.,* 2011, Cote *et al.,* 2010, Fokidis *et al.,*, 2012). An inability of the Robben Island African penguin adults to meet the energy requirements of their growing chicks might have caused HPA axis hyperactivation. However, we found no significant difference in CCI between chicks of the three different study sites, even though CCI is responsive to food availability (Campbell *et al.,* 2019). As such, food intake might not be a primary driver of GC production in this study, but this requires further research to confirm. Moreover, other factors not monitored during this study, such as differences in the presence of naturally occurring predators (Scheuerlein *et al.,* 2001; Sheriff *et al.,* 2011), pollution (Peakall *et al.,* 1981) and extreme weather events (Boersma and Rebstock 2014) can also be important drivers of the physiological stress response and could have been responsible for the varied ufGCM levels observed. Since the nesting habitat at the two sites is different, this could also have resulted in different levels of exposure to extreme weather events. However, additional research is required to determine which, if any, of the above-mentioned factors are important drivers of African penguin chick adrenocortical activity.

## Conclusion

This study monitored baseline ufGCM concentrations of African penguin chicks at sites with various levels of human disturbance. The results show that ufGCM concentrations differ between sites. Habituation, desensitization, exhaustion and/or life history stage may be important drivers of African penguin chick physiology in the face of human activity, even when stressors occur at comparatively low frequencies. Managers need to be mindful of minimizing activity at colonies with high human activity in the event that habituation is masking exhaustion, for example. However, future research should consider additional environmental factors, both anthropogenic and natural, as potential drivers of stress physiology in African penguin chicks. In addition to this, the effect of site-specific factors on adrenocortical activity should be considered. For ongoing conservation purposes, it would also be useful to monitor the stress responses of various age classes in this species, as stress physiology may change during development. Finally, as unhabituated chicks showed significantly elevated ufGCM levels compared to chicks experiencing medium and high levels of anthropogenic presence, managers and legislation should attempt to minimize all forms of activity (including tourism and construction) around important breeding colonies that are not regularly exposed to tourism.

## Funding

This work was supported by the Department of Biodiversity and Conservation Biology at the University of the Western Cape, the South African National Research Foundation (to G.W.M.) and the Earthwatch Institute (to R.B.S.).
